# Characterizing Sounds of Different Sources in a Commercial Broiler House

**DOI:** 10.3390/ani11030916

**Published:** 2021-03-23

**Authors:** Xiao Yang, Yang Zhao, Hairong Qi, George T. Tabler

**Affiliations:** 1 Department of Animal Science, The University of Tennessee, Knoxville, TN 37996, USA; xyang45@vols.utk.edu; 2 Department of Electrical and Computer Engineering, The University of Tennessee, Knoxville, TN 37996, USA; hqi@utk.edu; 3 Department of Poultry Science, Mississippi State University, Mississippi State, MS 39762, USA; ttabler@poultry.msstate.edu

**Keywords:** poultry, acoustic, audio, frequency, behavior

## Abstract

**Simple Summary:**

Acoustic signal in commercial broiler houses is a mixture of sounds from different sources. However, the characteristics of sounds from different sources have not been well understood. In this study, the sound frequency ranges of six common sounds, including bird vocalization, fan, feed system, heater, wing flapping and dustbathing, were determined; and their relations with bird age were investigated. The outcome of this research provides valuable information for using sound signal to monitor animal behavior and equipment operation.

**Abstract:**

Audio data collected in commercial broiler houses are mixed sounds of different sources that contain useful information regarding bird health condition, bird behavior, and equipment operation. However, characterizations of the sounds of different sources in commercial broiler houses have not been well established. The objective of this study was, therefore, to determine the frequency ranges of six common sounds, including bird vocalization, fan, feed system, heater, wing flapping, and dustbathing, at bird ages of week 1 to 8 in a commercial Ross 708 broiler house. In addition, the frequencies of flapping (in wing flapping events, flaps/s) and scratching (during dustbathing, scratches/s) behaviors were examined through sound analysis. A microphone was installed in the middle of broiler house at the height of 40 cm above the back of birds to record audio data at a sampling frequency of 44,100 Hz. A top-view camera was installed to continuously monitor bird activities. Total of 85 min audio data were manually labeled and fed to MATLAB for analysis. The audio data were decomposed using Maximum Overlap Discrete Wavelet Transform (MODWT). Decompositions of the six concerned sound sources were then transformed with the Fast Fourier Transform (FFT) method to generate the single-sided amplitude spectrums. By fitting the amplitude spectrum of each sound source into a Gaussian regression model, its frequency range was determined as the span of the three standard deviations (99% CI) away from the mean. The behavioral frequencies were determined by examining the spectrograms of wing flapping and dustbathing sounds. They were calculated by dividing the number of movements by the time duration of complete behavioral events. The frequency ranges of bird vocalization changed from 2481 ± 191–4409 ± 136 Hz to 1058 ± 123–2501 ± 88 Hz as birds grew. For the sound of fan, the frequency range increased from 129 ± 36–1141 ± 50 Hz to 454 ± 86–1449 ± 75 Hz over the flock. The sound frequencies of feed system, heater, wing flapping and dustbathing varied from 0 Hz to over 18,000 Hz. The behavioral frequencies of wing flapping were continuously decreased from week 3 (17 ± 4 flaps/s) to week 8 (10 ± 1 flaps/s). For dustbathing, the behavioral frequencies decreased from 16 ± 2 scratches/s in week 3 to 11 ± 1 scratches/s in week 6. In conclusion, characterizing sounds of different sound sources in commercial broiler houses provides useful information for further advanced acoustic analysis that may assist farm management in continuous monitoring of animal health and behavior. It should be noted that this study was conducted with one flock in a commercial house. The generalization of the results remains to be explored.

## 1. Introduction

Sound is defined as vibrations that travel through the air or another medium in the form of waves. The sound in commercial broiler houses is a mixture originated from multiple sources and can be generally categorized into two groups: (1) animal-based sounds and (2) equipment-based sounds. Animal-based sounds can be further categorized into animal vocalization (produced by the vibration of bird vocal cord) and behavioral sounds (produced during bird behaviors such as wing flapping and scratching). Equipment-based sounds refer to those produced during feed system, fans-on and heaters-on period.

Understanding the characteristics of sounds from different sources in broiler houses allows further sound analysis that may assist farmers in farm management and welfare monitoring. Frequency is one of the most important characteristics of sound. Audio signal in time domain only reflects the loudness, therefore, most of audio signal processing techniques and algorithms involve frequency analysis using techniques such as Fourier Transform [1], filtering [2], spectrogram [3], etc., for source identification.

In recent years, sound analysis as a non-invasive method has become an increasingly important tool in animal disease detection, behavior monitoring and welfare determination [4,5,6]. Cuan et al. [7] proposed a sound recognition method based on convolutional neural network to detect the infection of avian influenza, yielding 90% accuracy. Chung et al. [8] found that audio data can accurately detect (94% accuracy) and recognize (91% accuracy) pig wasting diseases. A sound-based product (SoundTalks NV., Leuven, Belgium) was commercialized to continuously and automatically detect pig respiratory disease at an early stage [9]. For animal behaviors, several studies were conducted to identify the feeding behavior of broiler chickens by analyzing audio data [10,11]. It has also been shown that peak frequency of bird vocalization can serve as the indicator of broiler age [12] and body weight [13].

In previous studies, the equipment-based sounds that are produced by mechanical systems were mostly considered as noise. The negative effects of farm noise on animal welfare have been reported [14,15,16]. However, as unavoidable acoustic sources in conventional broiler houses, equipment-based sounds also contain important information that can be used for farm management. For instance, the sound of the feed system can be an indicator for proper operation of the feed system, and the sound of fans for proper ventilation. On the other hand, understanding the characteristics of the equipment-based sounds may better help to remove these background sounds when only animal-based sounds are the concerns.

The objective of this study was to determine the frequency ranges of six common sounds, including bird vocalization, fan, feed system, heater, wing flapping and dustbathing in a commercial broiler house over an eight-week production cycle. In addition, the frequencies of flapping (in wing flapping events, flaps/sec) and scratching (during dustbathing, scratches/sec) behaviors were examined through sound analysis.

## 2. Materials and Methods

### 2.1. Housing, Animals and Management

The study was conducted in a commercial broiler house (east–west) located at Mississippi State University during May–June 2020. The house measured 120 × 13 × 3 m (L × W × H) with a capacity of 13,700 Ross 708 straight run broilers and a production cycle of eight weeks. The average slaughter body weight was 4.25 kg. All chicks were provided by a contract integrator in Mississippi. Flock management and diets followed the typical procedures in the industry. The lighting schedule was set to 24 L:0 D from 1 d to 7 d, 20 L:4 D from 8 d to 56 d. The light intensity was set to 54 lux from 1 d to 13 d, then gradually dimmed to 3 lux by 20 d and kept at 3 lux till 56 d. Lights were turned on at 05:00 h and turned off at 01:00 h of the next day. A total of 10 consistent speed fans (Acme BDR48J-A, 48”, Acme Engineering & Manufacturing Corp., Muskogee, OK, USA) were installed in the broiler house, with six fans across the east end wall, two fans on the north side wall, and two fans on the south side wall. The air flow rate was 34,660 m^3^/h at the static pressure of 0.05. The number and running time of fans were controlled by the house controller based on indoor air house temperature. As the experiment was conducted in summer, all fans were used by the end of the flock. A total of 13 heaters (Hog Slat GRO40, direct spark ignition, natural gas, 11.7 kW, Hog Slat Inc., Newton Grove, NC, USA) were distributed across the house and were installed 1.8 m above the floor. Heater on/off was controlled by the house controller as well. Automatic feeding system (Chore-Time, Revolution, A Division of CTB, Inc., Milford, IN, USA) was installed in the house. Feed was delivered along the feed line by an open coil auger inside the feed line tube and dropped into feeder pans halfway.

### 2.2. Audio Data Collection and Camera System

**Figure 1:**
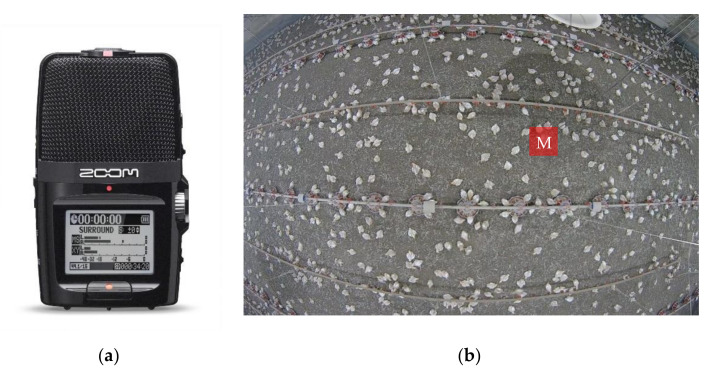
Image of the recorder (**a**) and location of the recorder M (**b**).

An H2n handy recorder (Zoom North American Inc., Hauppauge, NY, USA) (Figure 1a) was installed in the middle of broiler house at the height of 40 cm above the back of birds, as shown in Figure 1b. The recorder was 67.6 × 113.85 × 42.7 mm (W × H × D) in size and 130 g in weight. It was powered by two AA batteries or by an AD-17 USB to AC adapter (used in this study). Up to 120 dB sound can be captured. The sampling frequency was set to 44,100 Hz in this study. Audio signal was continuously recorded and saved to a 2 GB micro SD card and exported weekly.

A fisheye IP camera (Dahua, IPC-EW4431-ASW, Dahua Technology USA Inc., Irvine, CA, USA) was installed on the ceiling (height = 3 m) right above the microphone to monitor the wing flapping and dustbathing behaviors of broilers. The frame rate of the camera was 25 frames per second.

### 2.3. Sound Discription

Six specific types of sound signals, including bird vocalization, fan, feed system, heater, wing flapping and dustbathing, were examined in this study. The sound of the fans was produced when mixing fans and/or ventilation fans were working. As the microphone was installed around 50 m away from the fan, the sound of the fan was identified as the sound of wind from the recorded audios. The sound of the feed system was produced during the period when feeder augers were running for feed delivery. The sound of the heaters was produced during the first week when heaters were operating. Wing flapping was identified as a bout of continuous, rapid flapping behaviors [17]. Dustbathing was defined as birds performing classic vertical wing shakes, and performing side-rubs or prone leg scratches [18]. In this study, dustbathing specifically refers to the behavior of scratching the litter.

### 2.4. Audio Signal Labeling and Pre-Processing

**Figure 2:**
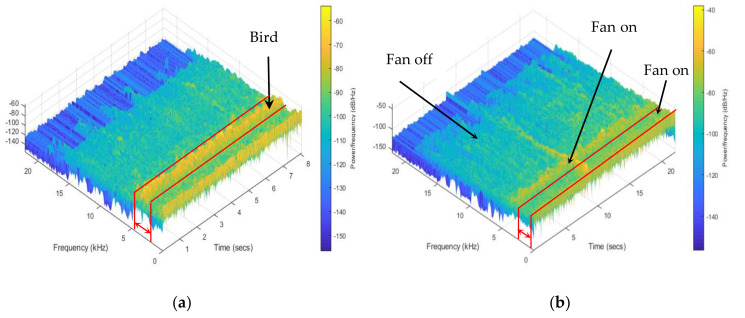

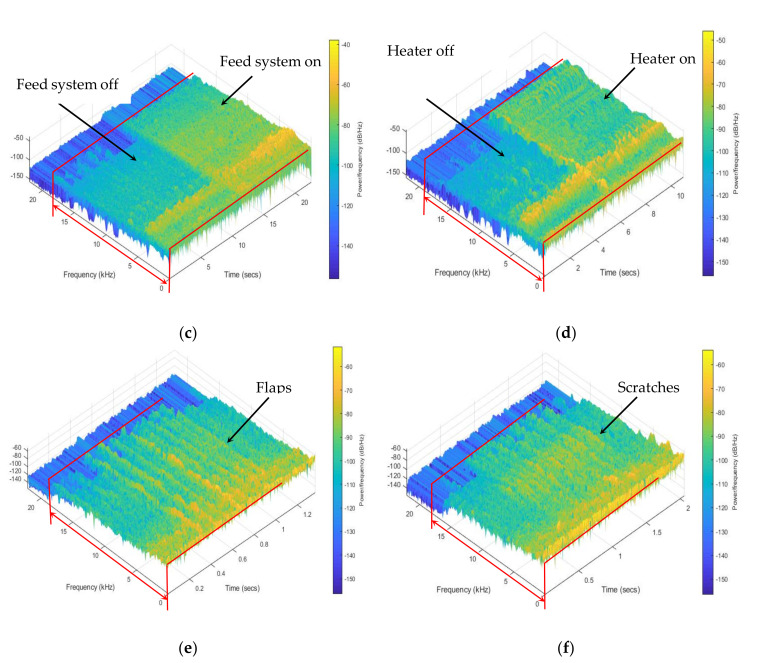
Example signals of bird vocalization (**a**), fan (**b**), feed system (**c**), heater (**d**), wing flapping (**e**) and dustbathing (**f**) in frequency domain.

The software Audacity (v.2.4.2, Audacity Team, GNU General Public License (GPL)) was used to visualize and identify the signal by comparing with the recorded videos. A summary of labeled data is shown in Table 1. Bird vocalization, fan, feed system and heater were trimmed into multiple 10-s clips. As the wing flapping and dustbathing usually lasted for a short time, behavioral data were trimmed into multiple 1-s clips. Audio clips were then fed into MATLAB (2018b, The MathWorks, Inc., Natick, MA, USA) for further analysis. Spectrograms were used for an initial visual check on frequency ranges, as shown in Figure 2. The frequencies of bird vocalization (Figure 2a) and fan (Figure 2b) ranged 0–5000 Hz. Sounds frequencies of the feed system (Figure 2c), heater (Figure 2d), wing flapping (Figure 2e) and dustbathing (Figure 2f) ranged 0–19,000 Hz.

### 2.5. Maximum Overlap Discrete Wavelet Transform (MODWT)

**Figure 3:**
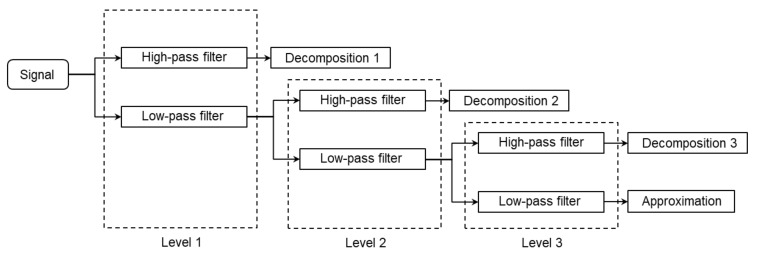
Flowchart for three-level Maximum Overlap Discrete Wavelet Transform.

**Figure 4:**
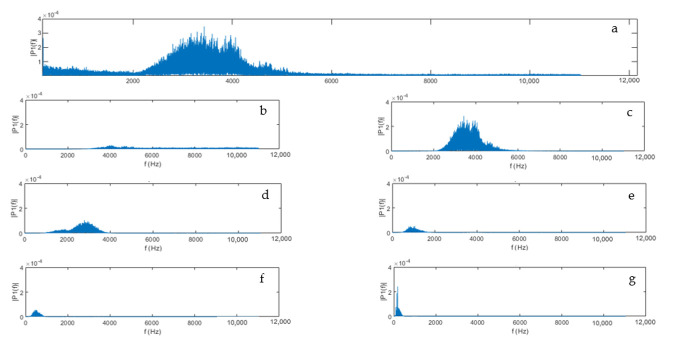
Single-sided amplitude spectrum of original signal (**a**) and decompositions 1–6 (**b**–**g**). Decomposition 2 (**c**) refers to bird vocalization, decomposition 4 (**e**) refers to fan.

The MODWT is a linear filtering operation that transforms a signal into multilevel wavelet and scaling coefficients [19]. As the flowchart for three-level MODWT shown in Figure 3, the MODWT applies low/high-pass filters to split the frequency components of the input signal into different scales. The filters are determined depending on the mother wavelet, which is selected in advance. The mother wavelets include Daubechies wavelet, Coiflects wavelet, Haar wavelet, and Symlet wavelet, and so on. Details on different wavelets can be found in publications by Percival and Walden [20]. The default mother wavelet (Symlet, N = 4) in MATLAB was used in our study. The decomposition level was determined based on the Equation (1) [21].

(1) $$L=int\left[log\left(n\right)\right]$$

where *int*[] is the function that returns the nearest integer of a number and *n* is the data length. For the study, *n* = 441,000, *L* = 6. The decompositions of bird vocalization signals are shown in Figure 4.

### 2.6. Signal Processing

**Figure 5:**
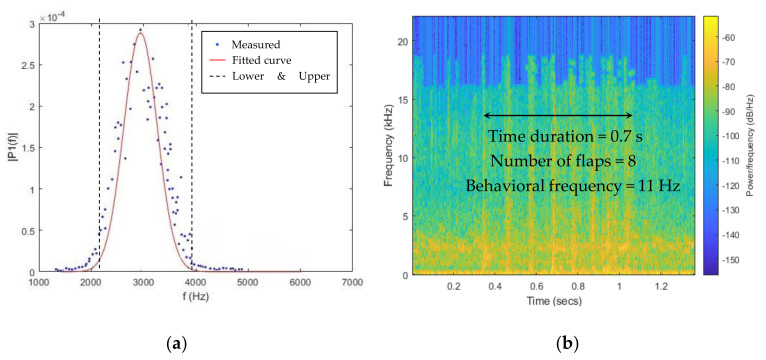
Example of Gaussian regression (**a**) and calculation of behavioral frequency (**b**). The sound sources of two sounds are bird vocalization (**a**) and wing flapping (**b**), respectively.

Two sounds with narrow frequency ranges (bird vocalization and fan) were decomposed using MODWT. Decompositions of both sounds were then transformed with the Fast Fourier Transform (FFT) method to generate the single-sided amplitude spectrums. By fitting the amplitude spectrum of each sound source into a Gaussian regression model, its frequency range was determined as the span of the three standard deviations (99% CI) away from the mean (Figure 5a). For those sounds with wide frequency ranges (feed system, heater, wing flapping and dustbathing), the upper ranges were determined by reading the largest frequency value from the FFT plot. The behavioral frequencies were determined by examining the occurrence of peak amplitudes in spectrograms during wing flapping and dustbathing (Figure 5b). They were calculated by dividing the number of wing flaps and scratches by the time duration of complete behavioral events.

### 2.7. Statistic Analysis

The effects of bird age on each different sound frequency range were analyzed using the PROC GLM (generalized linear model) procedure in SAS 10.9 (SAS Institute., Cary, NC, USA). The differences in behavioral frequency between wing flapping and dustbathing were also tested. A significant difference in multiple comparisons of group means was defined as *p* < 0.05.

## 3. Results

### 3.1. Bird Vocalization

Table 2 shows the lower and upper frequency limits of bird vocalization at different ages. Over the flock, the highest bird vocalization frequency was 4409 Hz, and the lowest was 1058 Hz. In general, both lower and upper limits continuously decreased as the bird grew, but the decrease was not linear. Frequencies dropped faster in the first few weeks. From week 1 to 4, the lower and upper limits decreased 1020 Hz and 1665 Hz, respectively. From week 4 to 8, the lower and upper limits decreased 403 Hz and 292 Hz, respectively. The frequency ranges during the first three weeks were larger than week 4 to 8.

### 3.2. Fan

Table 3 shows the upper and lower frequency limits of the fan at different bird ages. Generally, the lower and upper frequency limits of the fan increased as the bird grew. From week 1 to 8, the highest sound frequency of the fan was 1203 Hz, and the lowest was 305 Hz. In weeks 5–8, the lower frequency limits were significantly higher than other weeks. No significant difference was found in upper limits in weeks 2–4. The upper limits of weeks 6–8 were significantly higher than other weeks. The frequency range varied between 716 Hz (week 1) and 791 Hz (week 3).

### 3.3. Upper Limits of Feed System, Heater, Wing Flapping and Dustbathing

Table 4 shows the upper frequency limits of feed system, heater, wing flapping and dustbathing at different bird ages. In general, the frequency ranges of all the above sounds spanned widely from 0 to 19,000 Hz. For the feed system, the upper limits in the first two weeks were significantly lower than other weeks. In addition, the upper limit in week 8 was significantly higher than other weeks. The upper limit of the heater was the highest among the four types of sound. For wing flapping, the highest upper limit was observed in week 8, and the lowest was found in week 5. For dustbathing, the upper limit in week 3 was significantly lower than weeks 4–6. No significant difference was observed among weeks 4–6. When comparing two behavioral sounds, the average upper limit of wing flapping was higher than that of dustbathing.

### 3.4. Behavioral Frequency

Table 5 shows the behavioral frequencies of wing flapping and dustbathing at different bird ages. The behavioral frequencies of wing flapping and dustbathing were continuously decreased as the bird grew. In week 3, the behavioral frequencies of both wing flapping and dustbathing were significantly higher than other weeks. No significant difference was observed among weeks 4–6 for wing flapping and dustbathing. The behavioral frequency of wing flapping was significantly higher than that of dustbathing in weeks 4–6 (*p* = 0.049, *p* = 0.0003 and *p* = 0.0001, respectively).

## 4. Discussion

The frequency range of bird vocalization continuously decreased as the birds got older (Table 2). The result is consistent with that previously reported by Fontana et al. [22], who found a negative relationship between broiler vocalization frequency and bird age. The key assumption underlying the result is that larger animals often produce vocalizations with lower frequency than smaller animals. Vocalizations can be simply described as the result of tissue vibrations generated by the passage of air through a constriction in an animal’s vocal tract [23]. Due to the physical and energetic constrains, animals cannot efficiently produce sound waves larger than the size of their body or their sound-producing apparatus [24]. According to the theory, bird vocalization has been used to automatically and continuously monitor broiler body weight [13].

Different frequency-based filters have been adopted to remove the ventilation noise at pre-processing stage [12,25,26]. However, the frequency of sound produced by fan operation in commercial broiler houses remains to be understood. In this study, we found that the upper frequency range of the fan in commercial broiler houses varied between 1069–1203 Hz, which is slightly higher than those reported (1000 Hz) in previous studies [13,25]. Furthermore, our results show that both lower and upper limits of fan sound frequency generally increased as the birds grew, probably due to the increased ventilation rate and air speed. As birds got older and the weather got hotter, more fans were required for higher ventilation rates and air speed to exhaust excess heat production by birds. If the size of air inlets does not change accordingly to maintain a proper static pressure (e.g., 25 Pa), the airflow going through each fan will alter, which may affect the air interaction with fan blades and the fan sound frequency.

The frequency of feed system and heater ranged from 0 to 19,000 Hz. The result indicates that the sound produced during feed system and heater operation cannot be simply removed by adding a bandpass filter. Other noise reduction methods will be needed. There was statistical difference in the upper limits of the automatic feeder sounds among weeks, which was possibly due to the differences in feed particle sizes that affected the frictions between augers and feed tubes during feed delivery. However, the changes of the upper limits were small.

Acoustic signal can be a useful tool for learning animal behaviors. Microphones have been widely used to detect the animal behaviors, e.g., foraging of beef cattle [27,28], chewing of dairy cows [29], and feeding behavior of broilers [10]. However, using audio signal to determine broiler wing flapping and dustbathing behaviors has not been reported before. Our results showed the upper frequency range limits of these two behavioral sounds varied between 18,770–18840 Hz. Therefore, in order to avoid information loss of the signal, adding a filter < 19,000 Hz is not recommended before analyzing wing flapping and dustbathing behaviors at pre-processing. From the spectrograms, both wing flapping and dustbathing showed unique patterns in time series, which may provide valuable information for behavior classification and recognition in future.

During the first two weeks, wing flapping and dustbathing behaviors can be observed in recorded videos; however, they were not efficiently captured by the microphone. This indicates the limitation of using a microphone to learn the behaviors of young chicks. No dustbathing was observed in weeks 7–8. Similar results were reported by Meluzzi et al. [30], that dustbathing activity was decreased as broilers get older. Litter quality could be one of the factors. Broilers prefer to bathe at the area with loose and dry materials [31], while the litter is stiffer and moister at the end of flock. The behavioral frequency of wing flapping and dustbathing continuously decreased as birds grew. The possible reason is that older birds were more physically challenged to perform these behaviors due to the body weight. This hypothesis also indicates that the behavioral frequency could be another indicator of bird age. As no study has been conducted on broiler behavioral frequency so far, further investigation will be needed for the hypothesis.

As two types of non-invasive methods in animal research, cameras and microphones were often used separately. Both of them have proved to be efficiently deployed in precision livestock farming. It would be exciting if the two methods can be combined. Most off-the-shelf cameras come with both channels of video and audio, which can provide the system’s eyes and ears. Endless possibilities could be achieved by adding a computer as the brain, which will eventually achieve smart broiler farming.

## 5. Conclusions

In this study, the sound frequency ranges of bird vocalization, fan, feed system, heater, wing flapping and dustbathing in a commercial Ross 708 broiler house at different bird ages were determined using signal processing. The behavioral frequencies of wing flapping and dustbathing were examined as well. We concluded that the frequency range of bird vocalization continuously decreased as birds grew. The sound frequency of the fan generally increased from week 1 to week 8. The upper frequency range of the feed system, heater, wing flapping and dustbathing exceeded 18,000 Hz. Significant negative correlation of age on behavioral frequencies of wing flapping and dustbathing were observed. In summary, both broiler vocalization and equipment-based sounds showed temporal variations. These findings provide important insights into broiler welfare, health, and behavior determination using signal processing. Generalizing the results to other housing systems and broiler breeds will require more data.

## Figures and Tables

**Table 1:** Number of events and equivalent time labeled for each type of sound over eight weeks.

Sound Type	Total Time Labeled (min)	Total Number of Clips
Bird vocalization	40.0	240
Fan	40.0	240
Feed system	40.0	240
Heater	5.0	30
Wing flapping	6.7	402
Dustbathing	4.1	246

**Table 2:** Frequency range of bird vocalization at different bird ages (mean ± SD).

Bird Age (Week)	Lower Limit (Hz)	Upper Limit (Hz)
1	2481 ± 191 ^a^	4409 ± 136 ^a^
2	2038 ± 201 ^b^	4289 ± 89 ^b^
3	1889 ± 307 ^c^	3997 ± 128 ^c^
4	1461 ± 187 ^d^	2744 ± 155 ^d^
5	1418 ± 164 ^d^	2668 ± 114 ^e^
6	1190 ± 154 ^e^	2628 ± 113 ^e^
7	1100 ± 148 ^ef^	2615 ± 118 ^e^
8	1058 ± 123 ^f^	2501 ± 88 ^f^
average	1579	3231

^a,b,c,d,e,f^ Means in the same column with different superscripts are different (*p* < 0.05).

**Table 3:** Frequency range of fan at different bird ages (mean ± SD).

Bird Age (Week)	Lower Limit (Hz)	Upper Limit (Hz)
1	353 ± 62 ^c^	1069 ± 40 ^d^
2	331 ± 38 ^cd^	1080 ± 46 ^cd^
3	312 ± 40 ^d^	1103 ± 52 ^c^
4	335 ± 32 ^cd^	1101 ± 21 ^c^
5	407 ± 81 ^b^	1161 ± 78 ^b^
6	446 ± 28 ^a^	1191 ± 27 ^a^
7	417 ± 34 ^b^	1203 ± 36 ^a^
8	428 ± 44 ^a^	1200 ± 36 ^a^
average	379	1139

^a,b,c,d^ Means in the same column with different superscripts are different (*p* < 0.05).

**Table 4:** Upper frequency limits of feed system, heater, wing flapping and dustbathing at different bird ages (mean ± SD).

Bird Age (Week)	Feed System (Hz)	Heater (Hz)	Wing Flapping (Hz)	Dustbathing (Hz)
1	18,694 ± 149 ^c^	18903 ± 24	-	-
2	18,655 ± 244 ^c^	-	-	-
3	18,781 ± 61 ^b^	-	18,830 ± 27 ^ab^	18,771 ± 26 ^b^
4	18,819 ± 45 ^ab^	-	18,833 ± 50 ^a^	18,793 ± 27 ^a^
5	18,804 ± 54 ^ab^	-	18,819± 27 ^b^	18,791 ± 30 ^a^
6	18,813 ± 113 ^ab^	-	18,832± 34 ^ab^	18,797 ± 32 ^a^
7	18,833 ± 40 ^ab^	-	18,829 ± 27 ^ab^	-
8	18,857 ± 25 ^a^	-	18,837 ± 28 ^a^	-
average	18,782	18,903	18,830	18,788

^a,b,c^ Means in the same column with different superscripts are different (*p* < 0.05). Heater was only operated in week 1. The sound of wing flapping and dustbathing can be detected by microphone after week 2. No dustbathing behavior was identified in weeks 7 and 8.

**Table 5:** Behavioral frequency of wing flapping and dustbathing at different bird ages (mean ± SD).

Bird Age (Week)	Wing Flapping (Hz)	Dustbathing (Hz)
1	-	-
2	-	-
3	17 ± 4 ^Aa^	16 ± 2 ^Aa^
4	14 ± 3 ^Ab^	12 ± 1 ^Bb^
5	14 ± 3 ^Ab^	12 ± 2 ^Bb^
6	13 ± 2 ^Ab^	11 ± 1 ^Bb^
7	11 ± 2 ^c^	-
8	10 ± 1 ^c^	-

^a,b,c^ Means in the same column with different superscripts are different (*p* < 0.05). ^A,B^ Means in the same row with different superscripts are different (*p* < 0.05). The sound of wing flapping and dustbathing can be detected by microphone after week 3. No dustbathing was found on week 8.

## Data Availability

The data presented in this study are available on request from the corresponding author. The data are not publicly available due to restrictions by the collaborator broiler producer.
